# Role of the Polymer Microstructure in Controlling Colloidal and Thermo-Responsive Properties of Nano-Objects Prepared Via RAFT Polymerization in a Non-polar Medium

**DOI:** 10.1021/acs.langmuir.3c01065

**Published:** 2023-07-12

**Authors:** Gianmaria Gardoni, Nicolò Manfredini, Giulia Bagnato, Mattia Sponchioni, Davide Moscatelli

**Affiliations:** Department of Chemistry, Materials and Chemical Engineering “Giulio Natta”, Politecnico di Milano, Via Mancinelli 7, 20131 Milano, Italy

## Abstract

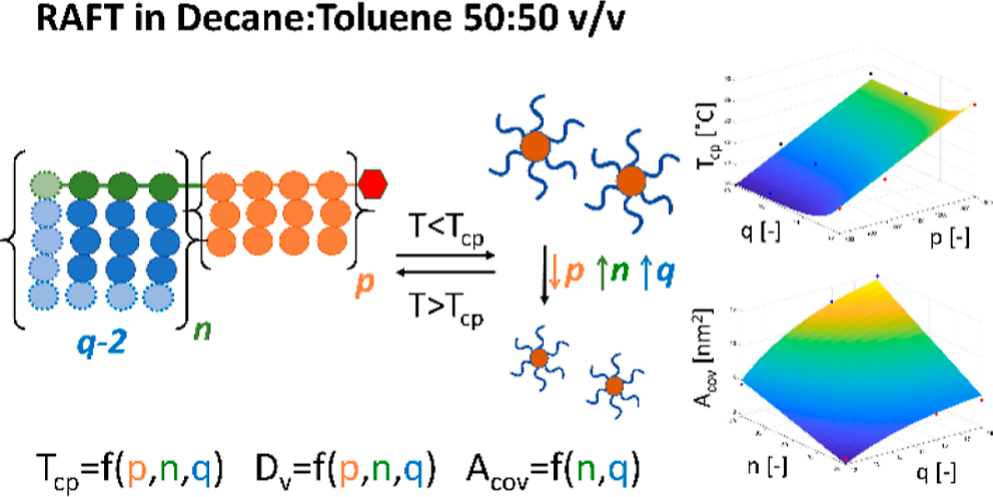

After having demonstrated their potential in biomedical applications, thermo-responsive block copolymers that are able to self-assemble into nano-objects in response to temperature modifications are becoming more and more appealing in other sectors, such as the oil and gas and lubricant fields. Reversible addition–fragmentation chain transfer (RAFT) polymerization-induced self-assembly has been demonstrated as a valuable strategy for producing nano-objects from modular block copolymers in non-polar media, required for the mentioned applications. Although the influence of the nature and size of the thermo-responsive block of these copolymers on the properties of the nano-objects is extensively studied in the literature, the role of the solvophilic block is often neglected. In this work, we elucidate the role of the main microstructural parameters, including those of the solvophilic portion, of block copolymers produced by RAFT polymerization in the hydrocarbon blend decane/toluene 50:50 v/v on the thermo-responsive behavior and colloidal properties of the resulting nano-objects. Two long-aliphatic chain monomers were employed for the synthesis of four macromolecular chain transfer agents (macroCTAs), with increasing solvophilicity according to the number of units (*n*) or length of the alkyl side chain (*q*). Subsequently, the macroCTAs were chain-extended with different repeating units of di(ethylene glycol) methyl ether methacrylate (*p*), leading to copolymers that are able to self-assemble below a critical temperature. We show that this cloud point can be tuned by acting on *n*, *p,* and *q*. On the other hand, the colloidal stability, expressed in terms of area of the particle covered by each solvophilic segment, is only a function of *n* and *q*, which provides a way for controlling the size distribution of the nano-objects and to decouple it from the cloud point.

## 1 Introduction

Reversible addition–fragmentation chain transfer (RAFT) polymerization proved to be a versatile technique for the production of polymers with a well-defined microstructure, reflected in a strict control over the properties of the final material.^1−3^ Owing to this precise control and possibility of accessing complex structures, RAFT polymerization is finding application in various sectors, spanning from biomedicine to oil and gas.^4−7^

In addition, RAFT polymerization is widely employed because of its versatility, which guarantees the synthesis of different polymers and copolymers with reduced interchain compositional drift in a wide range of solvents. Indeed, a quick look in the literature shows that RAFT has been successfully applied to the polymerization of different families of monomers (e.g., (meth)acrylates, (meth)acrylamides, and styrenes) in both polar and non-polar solvents.^8−12^ An additional advantage is the possibility of exploiting this technique under heterogeneous conditions, leading to nanostructured materials with controllable morphology. Because of this, RAFT has become one of the most employed pseudo-living polymerization for the production of polymer dispersions via polymerization-induced self-assembly (PISA).^13,14^

This has permitted to combine the amazing control over the copolymer microstructure offered by RAFT polymerization with the high polymerization rate offered by PISA in the formulation of highly concentrated latexes with well-defined properties, without the employment of surfactants or further post-processing like nanoprecipitation.^15−19^

Due to the predominant investigation of polymer nanoparticles in the biomedical field, the majority of the literature about the RAFT-mediated PISA refers to waterborne systems.^20−22^ However, in the last few years, there has been a rising interest in the synthesis of copolymer nanoparticles in non-polar solvents. Indeed, this would pave the way to nanostructured materials with tunable properties, particularly suitable as additives for oil blends like lubricants or for applications in the oil and gas sector, to be used in the upstream processes as w/o/w stabilizers.^23−27^ The literature available on this topic shows that with non-polar solvents also it is possible to efficiently synthesize well-defined block copolymers and to finely tune their microstructure by controlling parameters like chain length distribution and composition through a living polymerization technique, in analogy to what happens in polar media.^24,28−30^ The missing step is translating the wide research that has been conducted on the so-called waterborne smart materials, i.e., polymers that are able to respond to stimuli from the surrounding environment, to these non-polar media.^31^ Among these, thermo-responsive polymers that are able to sharply change their solubility in the solvent following temperature modifications are particularly appealing because of the ease of application of thermal stimuli. Indeed, thermo-responsive polymers have attracted considerable attention for controlled drug delivery and tissue engineering.^20,32^ At the same time, only few studies investigate thermo-responsive polymers synthesized in non-polar media, mainly discussing the influence of the thermo-responsive segment length on the phase separation.^27,29,33−38^

With the aim of covering this gap, in this work, we elucidate the role of key microstructural parameters of thermo-responsive copolymers in controlling the cloud point (*T*_cp_) of the formulation as well as other important colloidal properties, such as the morphology of the nano-objects formed below the *T*_cp_ and their hydrodynamic size. This systematic analysis is conducted on diblock copolymers comprising a solvophilic portion and a thermo-responsive segment with an upper critical solution temperature (UCST) and synthesized via RAFT polymerization in a mixture of decane and toluene 50:50 %v/v (dectol), representative of both the aliphatic and aromatic fraction of crude oil.^39,40^

**Scheme 1:**
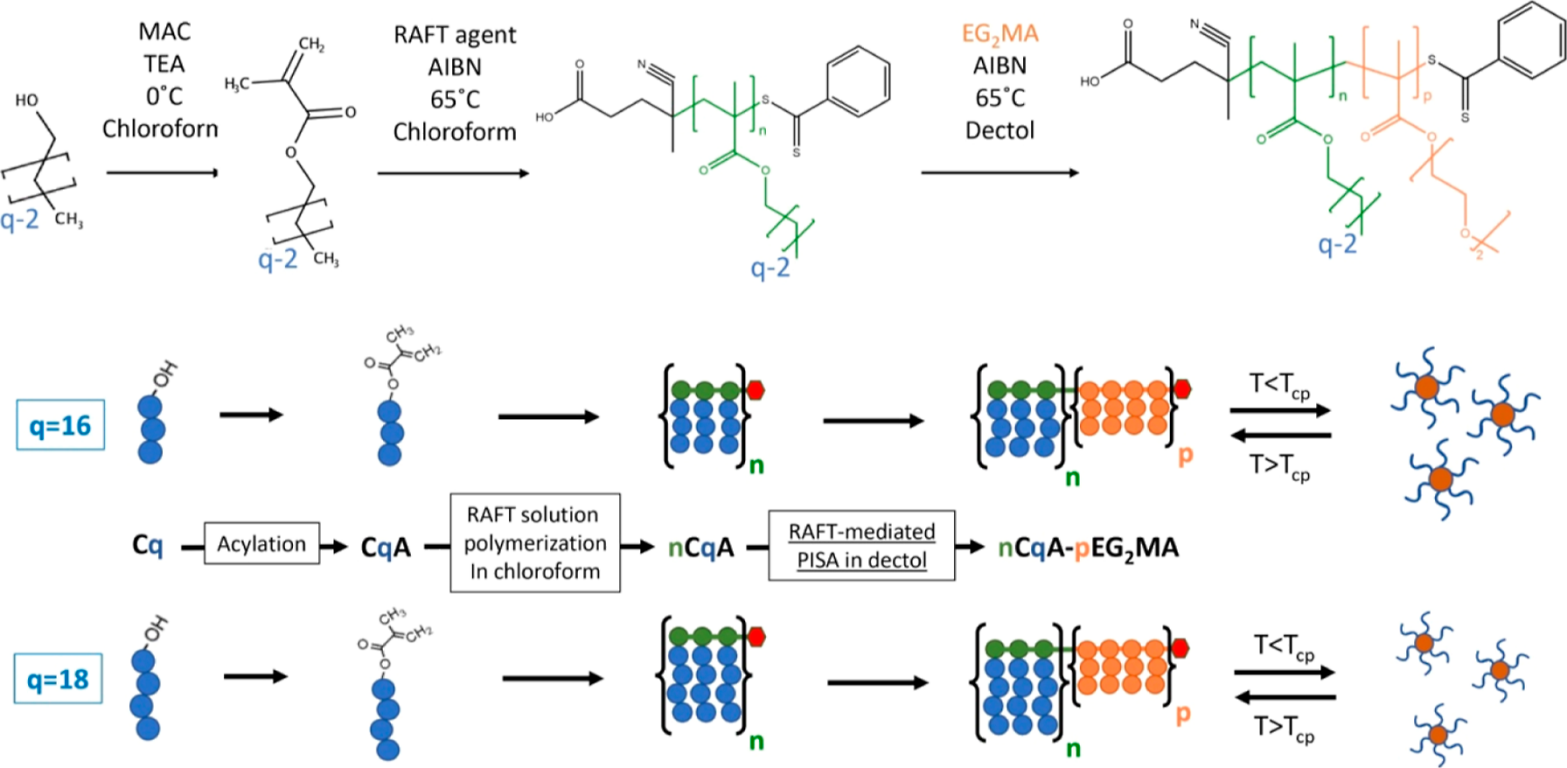
Schematic Representation of the Synthesis of Thermo-Responsive Nano-Objects with Four Degrees of Freedom, Namely, *q*, *n*, *p,* and Dry Content A three-step procure was followed: in the first step, acylation was used to synthesize long alkyl chain monomers, which were subsequently employed for the second and third step, i.e., RAFT solution and dispersion polymerization in organic solvent, respectively. More specifically, four macroCTAs with *n* = 20 and 40 were synthesized from monomers with different chain length (*q* = 16 and 18), represented in green. Each macroCTA was then chain-extended with EG_2_MA (orange) via RAFT dispersion polymerization, targeting *p* = 800, 1000, and 1400 while simultaneously changing the solid contents between 20, 30, and 40% w/w.

More specifically, as graphically depicted in Scheme 1, two solvophilic monomers with different aliphatic chain length (*q*) were obtained by acylation of the corresponding alcohols, i.e., 1-hexadecanol and 1-octadecanol, with methacryloyl chloride (MAC).^41^ Subsequently, the two monomers were polymerized via RAFT solution polymerization to produce oil-soluble macromolecular chain transfer agents (macroCTAs) with controllable chain length (*n*). Finally, these macroCTAs were chain-extended with di(ethylene glycol) methyl ether methacrylate (EG_2_MA), which shows a UCST in dectol as demonstrated in our previous work.^42^ The chain extension with a polymeric block showing a UCST leads to copolymers soluble in the non-polar solvent when the temperature is above the *T*_cp_ (tunable in the range 52–83 °C) and are able to spontaneously self-assemble into nano-objects when the temperature decreases below the *T*_cp_. By leveraging the good control provided by RAFT polymerization, different chain lengths were produced for this segment (*p*). This, combined with different solid contents during the synthesis, allowed obtaining a system with four easily controllable degrees of freedom, each one playing a specific role in the thermo-responsive behavior and the colloidal properties of the nano-objects formed from the self-assembly of these diblock copolymers.

Although the effect of the solid content and *p* is commonly studied when dealing with thermo-responsive polymers, we demonstrated that the often-neglected *q* and *n,* characteristics of the solvophilic portion, actually play a relevant role in determining the *T*_cp_, the morphology of the nano-objects formed in dectol, and the area covered by each stabilizer chain on the nanoparticle surface. This is particularly interesting since it adds a further way to modulate the characteristics of the formulation and to decouple *T*_cp_ and colloidal stability by acting on specific microstructural parameters.

## 2 Experimental Section

### 2.1 Materials

Decane (DEC, ≥99%, *M*_w_ = 142.28 g/mol, Sigma-Aldrich), toluene (TOL, ≥99.5%, *M*_w_ = 92.14 g/mol, Sigma-Aldrich), di(ethylene glycol)methyl ether methacrylate (EG2MA, *M*_w_ = 188.2 g/mol, Sigma-Aldrich), 2,2′-azobis(2-methylpropionitrile) (AIBN, ≥98%, *M*_w_ = 164.21 g/mol, Sigma-Aldrich), 1-hexadecanol (C16, *M*_w_ = 242.44 g/mol, Sigma-Aldrich), 1-octadecanol (C18, *M*_w_ = 270.49 g/mol, Sigma-Aldrich), methacryloyl chloride (MAC, ≥97%, *M*_w_ = 104.53 g/mol, Sigma-Aldrich), triethylamine (TEA, ≥99.5%, *M*_w_ = 101.19 g/mol, Sigma-Aldrich), Chloroform (CHCl_3_, ≥99%, *M*_w_ = 119.38 g/mol, Sigma-Aldrich), ethanol (EtOH , ≥99.8%, *M*_w_ = 46.07 g/mol, Sigma-Aldrich), 4-cyano-4-(phenylcarbonothioylthio) pentanoic acid (CPA, *M*_w_ = 279.38 g/mol, Sigma-Aldrich), tetrahydrofuran (THF, 99.9%, *M*_w_ = 72.11 g/mol, Sigma-Aldrich), chloroform-*d* (CDCl_3_, ≥99.8%, *M*_w_ = 120.38 g/mol, Sigma-Aldrich), deuterated dimethyl sulfoxide (DMSO-*d*_6_, ≥99.7%, *M*_w_ = 78.13 g/mol, Sigma-Aldrich), and deuterium oxide (D_2_O, 99.9%, *M*_w_ = 20.03 g/mol, Sigma-Aldrich). All chemicals were of analytical-grade purity and used as received unless otherwise noted.

### 2.2 Synthesis of the Oil-Soluble Monomers

Two lipophilic monomers were obtained by reacting primary alcohols, namely, C16 and C18, with methacryloyl chloride through a procedure already reported elsewhere.^41^

Taking as an example the case of acylated C16 (hereinafter C16A), 6.5 g of the alcohol (26.8 mmol) was added to a 250 mL round-bottom flask and mixed with 65 g of anhydrous CHCl_3_ and 4.07 g of TEA (40.2 mmol, i.e., 1.5 mol/mol with respect to C16).

The solution was stirred until the compounds were completely dissolved and cooled down in a water/ice bath. Then, 3.36 g of MAC (32.1 mmol, 1.2 mol/mol with respect to C16) was added dropwise by means of a syringe pump over 1.5 h. After the addition, the mixture was left to equilibrate to room temperature for 1 h. Subsequently, the mixture was filtered by means of a filter paper to remove the triethylamine hydrochloride salt and washed with HCl 0.1 M (1:1 v/v with respect to the filtered liquid) in a separating funnel. Eventually, the organic phase was withdrawn, dried under air, and characterized via proton nuclear magnetic resonance (^1^H NMR) on a Bruker Ultrashield 400 MHz spectrometer by dissolving 10 mg of the sample in 0.7 mL of CDCl_3_. The NMR spectra of the monomers and the equation used to determine the conversion of the alcohol can be found in the Supporting Information (see Figures S1, S2, and eq S1).

### 2.3 Synthesis of the Oil-Soluble MacroCTAs

The monomers synthesized were polymerized via RAFT solution polymerization to produce oil-soluble polymers with variable chain lengths. In particular, for each monomer, two macroCTAs were synthesized by setting the ratio between the monomer and CPA moles (*n* in Scheme 1) to 20 and 40. These four macroCTAs will be referred to as *n*C16A and *n*C18A.

As an example, to synthesize the 20C16A, 2 g of C16A (6.4 mmol) and 0.09 g of CPA (0.32 mmol, i.e., *n* = 20) were dissolved in 18 mL of chloroform (10% solid content) in a 50 mL round-bottom flask equipped with a magnetic stirrer and a condenser, to prevent evaporation of the solvent. The mixture was stirred until complete dissolution of the reactants and purged with nitrogen for 20 min. To initiate the reaction, 8 mg of AIBN (0.11 mmol, 1:3 molar ratio with respect to CPA) was dissolved in 1 mL of chloroform and added to the reaction mixture, which was left to react for 24 h at 65 °C in an oil bath. Summing the mass of initiator, CPA, and monomer, the total solid content was equal to 10% w/w. After the reaction, the polymer was purified by precipitation in an excess of EtOH (9:1 ratio in volume) and then centrifuged for 10 min at 5000 rpm. The final product was then recovered and dried under compressed air. Eventually, the polymers were recovered as viscous red liquids and stored at −20 °C.

The synthesized macroCTAs were characterized both via ^1^H NMR and gel permeation chromatography (GPC). For the NMR analysis, 10 mg of the product, both before and after purification, was dissolved in 0.7 mL of CDCl_3_. The analyses were performed on a Bruker 400 MHz spectrometer, with 64 scans per measurement. A representative ^1^H NMR spectrum with peak integration is shown in Figure S3 for 40C18A. The monomer conversion and degree of polymerization were calculated according to eqs S2 and S3. GPC was conducted on a Jasco LC-2000Plus apparatus, consisting of three styrene/divinylbenzene columns in series and a pre-column, coupled with a refractive index (RI) detector to record the signal. The columns had pore size of 10^3^, 10^5^, and 10^6^ Å, 300 mm length, and 8 mm internal diameter, while the pre-column had 50 mm length and 8 mm internal diameter. The samples were dissolved in THF with a concentration of 4 mg/mL and successively filtered with a 0.45 μm pore-size polytetrafluoroethylene (PTFE) membrane. The separation was done in THF as eluent at 35 °C with a flow rate of 1 mL/min. The molecular weight distribution (MWD) was determined through a calibration made with polystyrene standards (molecular weights from 580 to 3,250,000 Da, Polymer Laboratories). The chromatograms of the four macroCTAs produced are shown in Figure S4.

### 2.4 Synthesis of the Thermo-Responsive Block Copolymers

The synthesized oil-soluble macroCTAs were chain-extended with EG_2_MA to produce thermo-responsive block copolymers that are able to change their solubility in dectol, a 50/50% v/v mixture of decane and toluene, according to the external temperature. These diblock copolymers were synthesized at different molar ratios between EG_2_MA and the *n*C16A/C18A macroCTAs, generally denoted as *p*. In particular, *p* equal to 800, 1000, and 1400 were targeted for each macroCTA, with increasing solid content, from 20% up to 40% w/w. Hereinafter, these diblock copolymers will be referred to as *n*C16A-*p*EG_2_MA and *n*C18A-*p*EG_2_MA, to highlight the tailored process parameters. For example, to synthesize 20C16A-800EG_2_MA, 7.5 g of EG_2_MA (40 mmol) and 1.4 g of 20C16A (50 μmol, i.e., EG_2_MA/macroCTA = 800 mol/mol) were dissolved in 32 g of dectol in a 100 mL round-bottom flask equipped with a magnetic stirrer. The mixture was stirred until complete dissolution of the reactants and purged with nitrogen for 20 min. To initiate the reaction, 3 mg of AIBN (17 μmol, 1/3 molar ratio with respect to the macroCTA) was dissolved in 1 mL of dectol and added to the reaction mixture, which was left to react for 24 h at 65 °C in an oil bath. Once the reaction was completed, the samples were cooled in a controlled way by letting them equilibrate to room temperature in tap water.

For the characterization, the polymer was analyzed via ^1^H NMR and GPC with the procedures reported in Section 2.2. ^1^H NMR analysis, with a representative spectrum reported in Figure S5 for the sample 21C18A-800EG2MA, allowed the determination of the conversion of EG_2_MA (eq S4), degree of polymerization *p* (eq S5), and CTA efficiency (eq S6), while through GPC the MWD of the block copolymer was determined, together with the number-averaged molecular weight (*M*_n_), the weight-averaged molecular weight (*M*_w_), and polydispersity (Đ). These properties are summarized in Tables S1–S6.

Moreover, the dispersions were analyzed via dynamic light scattering (DLS) on a Malvern Zetasizer Nano ZS at a scattering angle of 173° to determine the cloud point (*T*_cp_), volume-average diameter (*D*_v_), and polydispersity index (PDI). For the analyses, the dispersions were diluted to 0.5% w/w in dectol, and the measurements were performed in triplicate. *T*_cp_ was determined by heating and cooling the samples in a particular temperature range (50–90 °C) and measuring the NP size and relative scattering intensity (RSI) every 1 °C with an equilibration of 5 min before each measurement. In particular, *T*_cp_ was determined as the inflection point in the size vs temperature curve. Eventually, for each chain-extended macroCTA, a phase diagram at temperature below the *T*_cp_, more precisely 25 °C, was built. Pictures of the morphologies in the various regions were acquired through transmission electron microscopy (TEM), using a Philips CM200 electron microscope at 200 kV equipped with a field emission gun filament. The samples were diluted to 0.1% w/w in dectol, and 30 μL of the dispersion, after being carefully mixed, was deposited onto a 200-mesh carbon-coated copper grid and dried. After drying, 2048 Å ∼2048 pixels images with 256 grey levels were recorded through a Gatan US 1000 CCD camera.

## 3 Results and Discussion

### 3.1 Oil-Soluble MacroCTAs with Controllable Length and Side-Chains

Well-defined diblock copolymers have been synthesized through a three-steps procedure in a non-polar solvent, as shown in Scheme 1.

In the first step, two aliphatic alcohols have been functionalized with a vinyl group in order to make them polymerizable through a radical process.

In particular, 1-hexadecanol (C16) and 1-octadecanol (C18) have been chosen because of their long hydrocarbon chains, responsible for the high lipophilicity and thus solubility in dectol, as well as for the presence of the terminal hydroxyl group, useful for the functionalization of the molecules through acylation. Indeed, C16 and C18 methacrylates (namely C16A and C18A) were synthesized from the respective alcohol and methacryloyl chloride. The products have been characterized in terms of alcohol conversion via ^1^H NMR. The spectra with peak integration are shown in Figure S1 for C16A and Figure S2 for C18A. The alcohol conversion was determined to be 93.6% for C16 and 88% for C18, as calculated through eq S1. After the synthesis, these monomers were polymerized via RAFT solution polymerization in chloroform to produce four different oil-soluble macroCTAs, which differ by the length of the brushes (*q* = 16 or 18) and the chain length (*n*, targeted either 20 or 40). In this way, it was possible to synthesize block copolymers with tailored stabilizing properties by carefully choosing the appropriate monomer and degree of polymerization, two of the four parameters considered in this work.

Through GPC and ^1^H NMR, the macroCTAs were characterized in terms of monomer conversion, degree of polymerization, average chain length, and molecular weight distribution. The NMR spectra and GPC chromatograms are reported in the Supporting Information (Figures S3 and S4), while the properties of the polymers are summarized in Table 1.

**Table 1:** Conversion (χ), *n*, Number-Average Molecular Weight (*M*_n_), Weight-Average Molecular Weight (*M*_w_), and Polydispersity (Đ) of the *n*C16A and *n*C18A macroCTAs

sample	χ (%)	*n* [-]	*M*_n_ [Da]	*M*_w_ [Da]	*Đ* [-]
20C16A	92	23	4120	4900	1.19
40C16A	86	43	6510	7550	1.16
20C18A	92	21	4280	5050	1.18
40C18A	92	39	8150	9290	1.14

In all the syntheses, high conversions of the monomers were obtained, as computed via ^1^H NMR according to eq S2. Moreover, the narrow molecular weight distributions (i.e., *Đ* <1.2) and the similarity between the actual and theoretical degree of polymerization (Table 1) confirmed once more the pseudo-living characteristics of the RAFT polymerization, which allows synthesizing the materials with well-defined features.

### 3.2 Characterization of the Diblock Copolymers

After having synthesized macroCTAs with controllable chain length, narrow molecular weight distribution, and different lengths of the brushes, these were chain-extended with EG_2_MA. The synthesis was conducted via RAFT-mediated PISA in dectol, a solvent representative of both the aliphatic and the aromatic components of crude oil, to form compartmentalized diblock copolymers with controllable microstructures.

In particular, we synthesized a library of diblock copolymers by independently varying four degrees of freedom, namely, *n* and *q* for the solvophilic block, *p* for the solvophobic block (i.e., 800, 1000, and 1400), and the solid content (i.e., 20, 30, and 40% w/w). GPC allowed the determination of the MWD of the products, while ^1^H NMR was used to determine monomer conversion and the actual degree of polymerization *p* according to eqs S4 and S5. An example of the ^1^H NMR spectrum is reported in Figure S5 for the sample 21C18A-800EG_2_MA, to show the peaks considered for the calculations. Eventually, the properties of all the diblock copolymers are summarized in Tables S1–S6 for each dry content and solvophilic macroCTA employed.

In all the syntheses, high EG_2_MA conversions >92% and actual *p* close to the targeted one have been achieved, confirming the possibility of adding to the solvophilic macroCTA the desired number of thermo-responsive repeating units and therefore modulate the microstructure of the final polymers.

**Figure 1:**
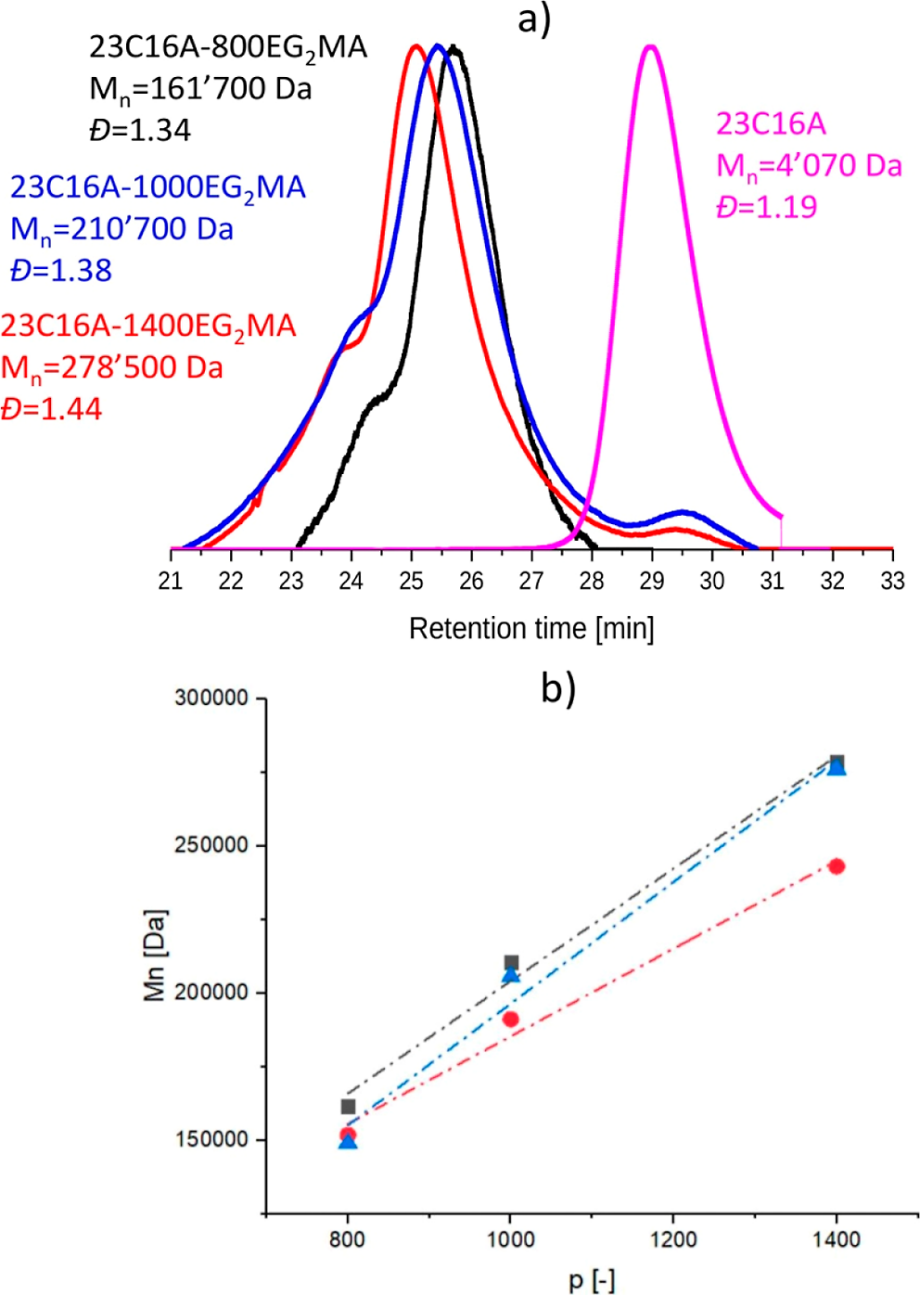
(a) GPC chromatograms for the 23C16A-pEG_2_MA 20% w/w copolymers. (b) Mn vs *p* for the 23C16A-pEG2MA 20% (black squares), 30% (red circles), and 40% (blue triangles) copolymers. The dashed lines represent the linear fits of the experimental data, with *R*^2^ = 0.991 (20% syntheses), *R*^2^ = 0.988 (30% syntheses) and *R*^2^ = 0.984 (40% syntheses).

Moreover, the GPC traces for the 23C16A-*p*EG_2_MA copolymers at 20% w/w shown in Figure 1a confirm the synthesis of well-defined diblock copolymers with high block efficiency and narrow MWDs. The small early-eluting shoulders present in the curves are due to some dimethacrylate impurities in the commercial EG_2_MA that lead to the formation of crosslinks, as previously reported by Armes et al.^43^ The presence of dimethacrylate impurities in the commercial monomer was also verified via HPLC, as shown in Figure S6.

The good control over the polymerization is further confirmed by the linear increase in the number-average molecular weight (*M*_n_) with *p*, as shown in Figure 1b for the copolymer 23C16A-*p*EG_2_MA 20% and in Figures S7–S9 for the cases with different degrees of polymerization *n* and different lengths of the solvophilic brushes (*q*). In fact, in the case of a pseudo-living polymerization, *M*_n_ is expected to grow with *p* according to eq 1.
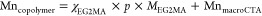
1where χ_EG2MA_ is the monomer conversion, *M*_EG2MA_ is the molecular weight of EG_2_MA, and Mn_copolymer_ and Mn_macroCTA_ are the number-average molecular weights of the final copolymer and of the macroCTA, respectively.

Overall, well-defined diblock copolymers could be synthesized in a non-polar solvent via RAFT dispersion polymerization. The outstanding control granted by RAFT polymerization allowed synthesizing diblock copolymers with different solvophilicities and microstructures by properly varying four independent parameters, namely, the lengths of the solvophilic block brushes *q*, the number of repeating units in the solvophilic block *n*, the degree of polymerization of the thermo-responsive block *p*, and the solid content.

### 3.3 Influence of the Copolymer Microstructure on the Nano-Object Properties

After having characterized the copolymer microstructure, their thermo-responsive behavior and ability to self-assemble into nano-objects with peculiar morphology and colloidal properties have been investigated in relationship to the copolymer structural parameters.

**Figure 2:**
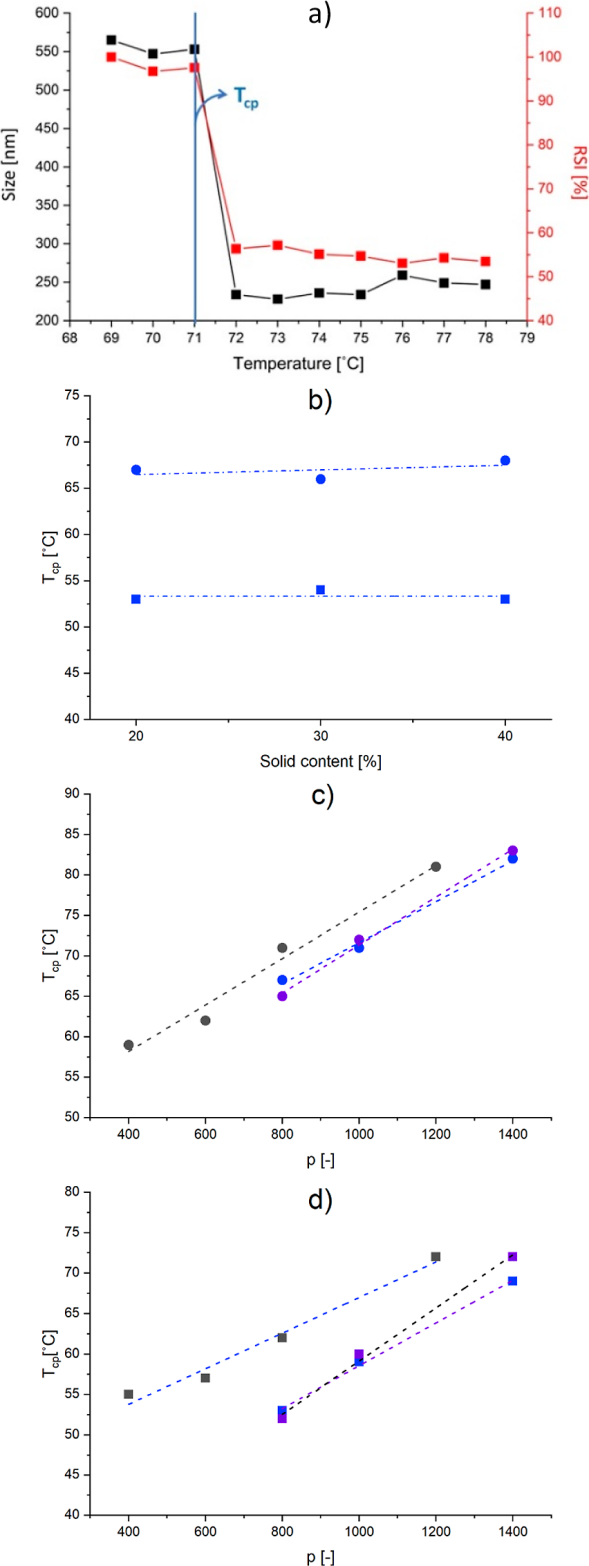
(a) Volume-average size and RSI for the copolymer 23C16A-1000EG_2_MA at 20% w/w. The graph shows how the cloud point (*T*_cp_) was determined for all the copolymers, namely as the temperature where both size and RSI sharply decrease. (b) Trend of *T*_cp_ with solid content for 23C16A-800EG2MA (*n* = 20, circles) and 43C16A-800EG2MA (*n* = 40, squares) (c) correlation between the *T*_cp_ and the degree of polymerization of the thermo-responsive block for the copolymers with *n* = 20 and variable *q* (black for *q* = 12, blue for *q* = 16, and purple for *q* = 18) and with (d) *n* = 40 and variable *q* (black for *q* = 12, blue for *q* = 16, and purple for *q* = 18). The dashed lines represent the function obtained by numerical data fitting according to eq 2. The data for the cases with *q* = 12 are reproduced from ref (42). Copyright 2023 American Chemical Society.

In particular, the thermo-responsive behavior is due to the poly(EG_2_MA) segment, which has a UCST-like behavior in dectol, as demonstrated in our previous work.^42^ Therefore, it is possible to synthesize block copolymers that are soluble in dectol above the *T*_cp_, while self-assembling into nano-objects when the temperature is decreased below this critical temperature. This behavior was confirmed for all the synthesized diblock copolymers via DLS by measuring the average size and relative scattering intensity (RSI) in a wide range of temperature, as it can be seen in Figure 2a for the sample 23C16A-1000EG_2_MA 20%, taken as an example. The RSI is a parameter that is proportional to the nano-object concentration and size at the sixth power,^44^ therefore it is useful in determining the change of solubility or aggregation of the polymer sample. In particular, its sharp decrease indicates the copolymer solubilization in the continuous phase. The *T*_cp_ of the copolymers was taken as the temperature where a steep decrease of both size and RSI happened, i.e., 71 °C in the case of the example in Figure 2a. The same procedure was followed for all the copolymers in order to be able to relate their cloud point to the corresponding solid content and structural parameters, more precisely *p*, *n,* and *q*. The *T*_cp_ measured for all the copolymers investigated are summarized in Table S7.

First, Figure 2b highlights that the solid content does not affect the cloud point of the copolymer, which is therefore only a function of the nature of the thermo-responsive monomer and the structural parameters of both the solvophilic and solvophobic block. Considering these parameters, from Figure 2c,d it can be noticed that RAFT polymerization allows synthesizing copolymers with *T*_cp_ in a wide range (from 52 to 83 °C). In particular, it is possible to observe that *T*_cp_ is a linear function of *p*, which therefore represents the main factor in determining the thermo-responsive behavior of these copolymers. This finding is indeed in line with what is already reported in other studies.^42,45−47^ On the other hand, the role played by the microstructure of the solvophilic block on the *T*_cp_ is never considered. The systematic investigation carried out in this work instead also allows elucidating the influence of the solvophilic parameters *n* and *q* on the copolymer cloud point. Indeed, comparing the behavior of samples with equal length of the thermo-responsive block but different degrees of polymerization of the solvophilic portion *n* (Figure 2c,d), it is possible to notice that the *T*_cp_ decreases moving from *n* = 20 to *n* = 40 due to the greater solubility of this portion of the copolymer, which hinders the copolymer self-assembly. As a matter of fact, for some of the samples synthesized with *n* = 40, the *T*_cp_ is below the reaction temperature. This brings as a consequence that the temperature-mediated self-assembly does not occur during the synthesis for these samples, and polymerization proceeds in solution, rather than in dispersion. The differences among the two mechanisms have not been investigated as they are out of the scope of this work. Indeed, they do not play a relevant role in determining the *T*_cp_ of the final copolymers, as this is only related to the polymer microstructure.^34,48^

The same effect is found when the length of the brushes of the solvophilic block (*q*) is increased. To better highlight the influence of this parameter, the results are complemented with the data from our previous work.^42^ In that case, thermo-responsive copolymers were synthesized employing lauryl methacrylate (LMA, *q* = 12) as stabilizer and EG_2_MA as thermo-responsive units and varying both *n* and *p* parameters. In this way, three distinct values of *q* are compared in this analysis, namely, 12, 16, and 18.

When both *n* and *p* are kept constant, the higher the number of carbon atoms in the stabilizing monomer the greater the solubility of the copolymer, which therefore requires a lower temperature to self-assemble. This effect is particularly visible when moving from *q* = 12 to longer alkyl side chains. On the other hand, no major differences were observed by using monomers with *q* = 16 and 18. This led to the hypothesis that the increase in solvophilicity with *q* reaches a plateau so that its effect on the final *T*_cp_ is negligible once a certain length of the chain is overcome (Figure 2c,d).

To better visualize the effect of the various structural parameters on the *T*_cp_ of the copolymer, the experimental data were fitted through a genetic algorithm in MATLAB. Exploiting the highlighted dependence of *T*_cp_ from the microstructural parameters of the copolymer, its behavior was analytically described according to eq 2
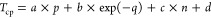
2where we hypothesized a linear dependence with *n* and *p* (already confirmed through the results above) and an exponential one with *q*, to account for the asymptotic behavior. The objective function that was minimized to determine the fitting parameters was the residual sum of squares (RSS, see eq 3).
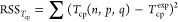
3where *T*_cp_(*n*,*p*,*q*) is the cloud point as a function of *n*, *p,* and *q* computed through eq 2, whereas *T*_cp_^exp^ is the experimental value measured for the same set of structural parameters.

The best set of fitting parameters is (0.028 1.01 × 10^6^ −0.565 54.553), leading to an .

**Figure 3:**
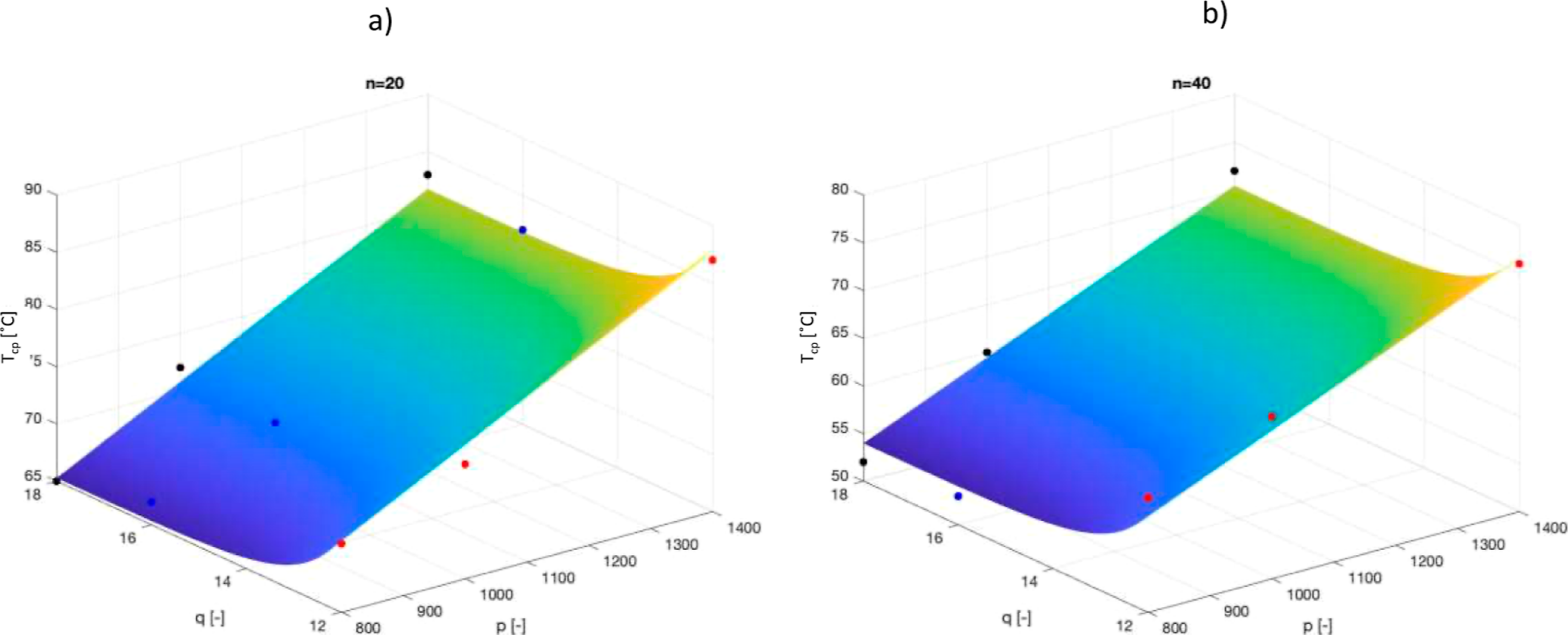
*T*_cp_ as a function of *p* and *q* for two different values of *n*, respectively, *n* = 20 (a) and *n* = 40 (b). The fit has been obtained through a genetic algorithm hypothesizing a linear dependence of *T*_cp_ with *p* and *n* and an exponential one with *q*.

With this, it was possible to determine the design space of *T*_cp_ as a function of *p* and *q* for *n* = 20 and *n* = 40 (Figure 3a,b, respectively). Given the nature of the optimization problem, it is worth highlighting that these results should not be extrapolated outside the range of parameters investigated in this work. However, inside these intervals, the predicted *T*_cp_ shows a good agreement with the experimental data (dashed lines in Figure 2c,d).

Therefore, by using the proposed model, it is possible to determine a priori the final thermo-responsive behavior of the copolymers according to their structural parameters *n*, *p*, and *q*. This analysis confirmed that, despite being scarcely investigated in the literature, the solvophilic block parameters play a relevant role in the definition of the thermo-responsive behavior of the system and their influence cannot be neglected when designing the copolymer for a specific application.

Once we confirmed the possibility of finely tuning the *T*_cp_ of the block copolymers by modifying the properties of both the solvophilic and solvophobic blocks via RAFT polymerization, we moved on to the analysis of the macroscopic behavior of the polymers.

Interestingly, the bulk response of the copolymer dispersions below their *T*_cp_ showed that it is influenced by all of the four parameters considered: *n*, *p*, solid content, and *q*. To have a better insight into this discovery, four phase diagrams have been created to unveil the dependence of the macroscopic appearance on all the copolymer structural parameters at a temperature below the *T*_cp_, taken as 25 °C for convenience. From a visual inspection, it was possible to determine four different macroscopic states of the formulation, namely, free-flowing liquid, viscous cloudy dispersion, self-standing clear gel, and precipitation of a polymer-rich phase from dectol. Pictures that show these four macroscopic behaviors are available in Figure S10.

The different shapes of the four phase diagrams confirm that the bulk behavior of the copolymer formulations is strictly connected to the four parameters considered.

First, it can be observed that the region with low solid content mainly behaves as a free-flowing liquid, which was associated with the formation of spherical nanoparticles, as disclosed by TEM (Figure 5a) and DLS. In fact, the low polydispersity indexes (PDI) measured via DLS and reported in Table S8 are indicative of isotropic morphologies and hence of spherical particles.

**Figure 4:**
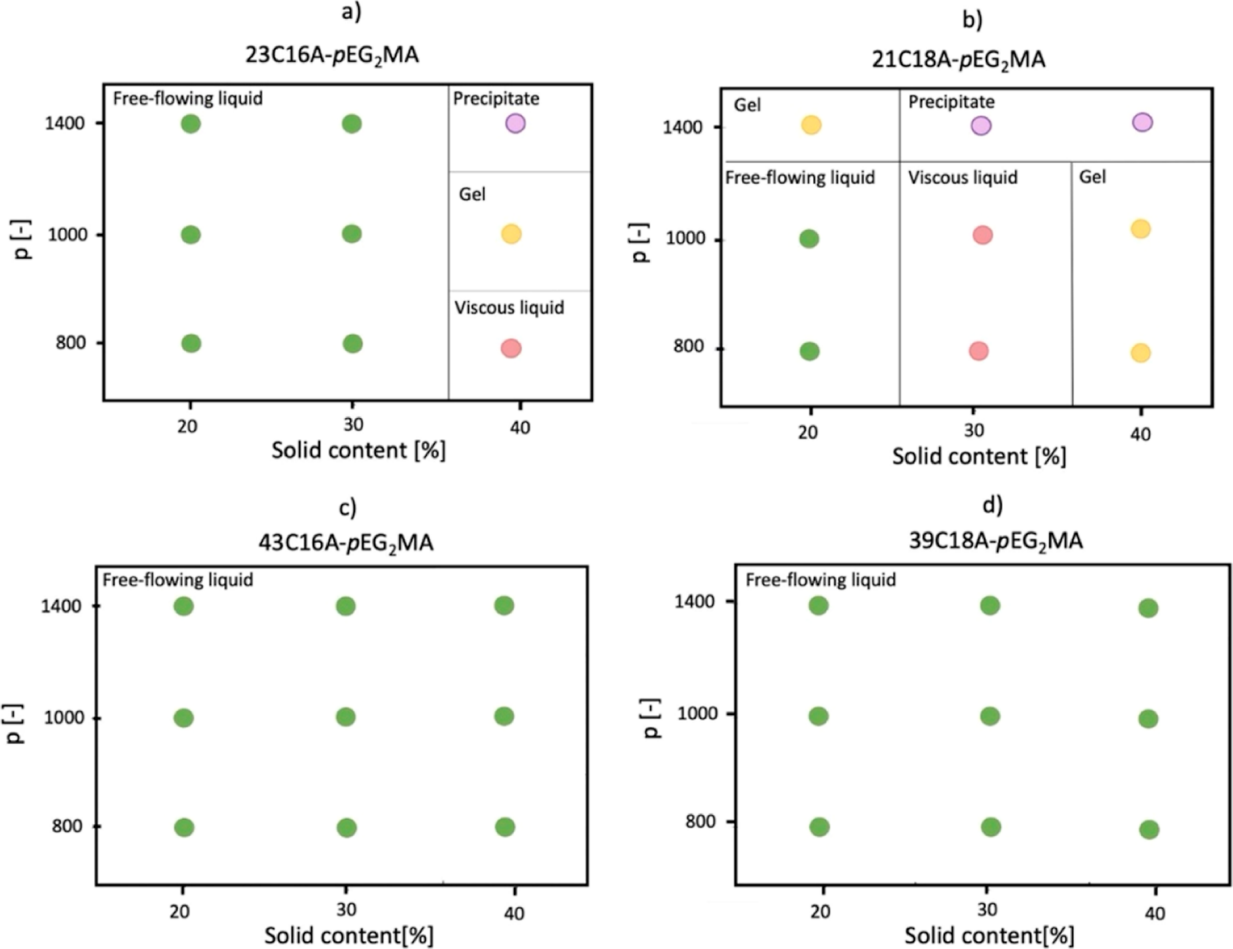
Phase diagrams for different samples at a fixed temperature of 25 °C and variable solid contents: (a) 23C16A-pEG_2_MA; (b) 21C18A-pEG_2_MA; (c) 43C16A-pEG_2_MA; (d) 39C18A-pEG_2_MA. Four different macroscopic behaviors are highlighted: free-flowing liquid (green dots), viscous liquid (red dots), gel (yellow dots), and precipitate (purple dots).

**Figure 5:**
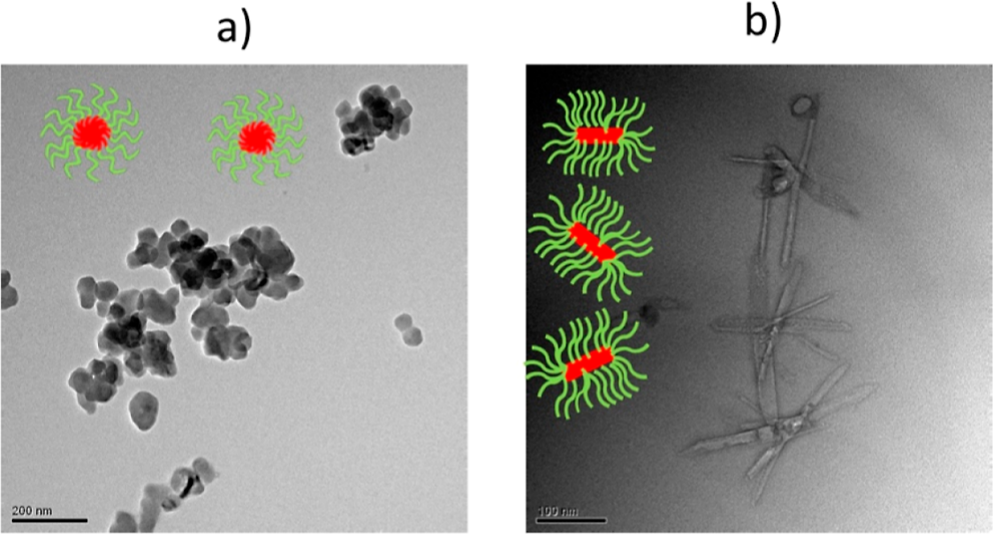
(a) TEM micrograph of the sample 23C16A-1000EG2MA at 20% w/w solid content, which shows a free-flowing liquid behavior (b) TEM micrograph of the sample 23C16A-1400EG2MA at 40% w/w solid content, which shows a self-standing gel behavior.

The extent of the region of spherical nanoparticles is expanded also to higher solid content in the case of copolymers with a longer solvophilic block (*n* = 40). This can be attributed to the greater stabilizing properties of this solvophilic portion, which reduces the packing parameter and favors the formation of morphologies with a high surface area. On the other hand, when the degree of polymerization is fixed at *n* = 20, at solid contents larger than 20%, the increased packing parameter causes the self-assembly into higher order morphologies. Indeed, the copolymers adopt more packed conformations such as rods, as seen in TEM images (Figure 5b), which we associate with gel appearance. In the end, when the block copolymer is too asymmetric and the solid content is high, the polymer precipitates forming a different phase from dectol.

Until now, the copolymer stability has been associated mainly with the length of the solvophilic block. However, in this work an additional degree of freedom is worth being considered, i.e., the length of the brushes of the solvophilic block, which is seldom considered.

From a comparison between copolymers made by solvophilic monomers with different *q*, it can be seen that, surprisingly, the macroCTA with *q* = 16 seems to have a greater stabilizing effect than the one with *q* = 18, as the free-flowing liquid behavior covers a wider region. This result can be ascribed to the formation of a copolymer with a higher crystallinity in the case of *q* = 18, which could introduce some destabilization during the rearrangement into nanoparticles.^49,50^ Nonetheless, this instability is lost when the solvophilicity of the copolymer is further increased by increasing *n*.

A more appropriate evaluation of the effect of the length of the brushes can be done by considering our previous work, where lauryl methacrylate (*q* = 12) was used as solvophilic monomer for the synthesis of two different macroCTAs with *n* equal to 20 and 40.^42^ These two stabilizing blocks, namely, 23LMA and 40LMA, were then chain-extended with *p* units of EG_2_MA, following a procedure similar to the one reported in this work. In this case, the shorter carbon chain (*q* = 12) leads to a lower solvophilicity and therefore a narrower free-flowing liquid region, which is restricted to the 20% solid content in both the cases of 23LMA and 40LMA. Moreover, taking into consideration values of *p* comparable to the one used in this work (*p* = 800 and 1000), it is possible to notice the formation of a viscous liquid/self-standing gel at lower solid contents. This finding is particularly important since it adds an additional degree of freedom that can be manipulated to obtain the desired final conformation.

After having highlighted the possibility of controlling the thermo-responsive behavior and of targeting different bulk responses by finely tuning the copolymer microstructure, we investigated the role of *n*, *q*, and *p* in the colloidal properties of the spherical nanoparticles formed at 25 °C, by focusing on the free-flowing liquid region that dominates the phase diagrams in Figure 4. Indeed, in this region the copolymers self-assemble into nanoparticles with different size according to the microstructure of both the solvophilic (*n* and *q*) and the solvophobic (*p*) blocks.

It is worth underlying that for all the samples, DLS revealed narrowly distributed nanoparticles (Table S8), with PDI < 0.2, further suggesting the presence of isotropic objects. From a purely geometrical model, considering spherical particles, the diameter *D*_v_ is expected to be a function of the constituting copolymer microstructure according to eq 4.^51^
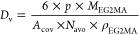
4where ρ_EG2MA_ is the density of the poly(EG_2_MA) block, *M*_EG2MA_ is the molecular weight of the EG_2_MA monomer, *N*_avo_ is the Avogadro number, and *A*_cov_ is the area on the nanoparticle surface covered by a single stabilizer chain.

**Figure 6:**
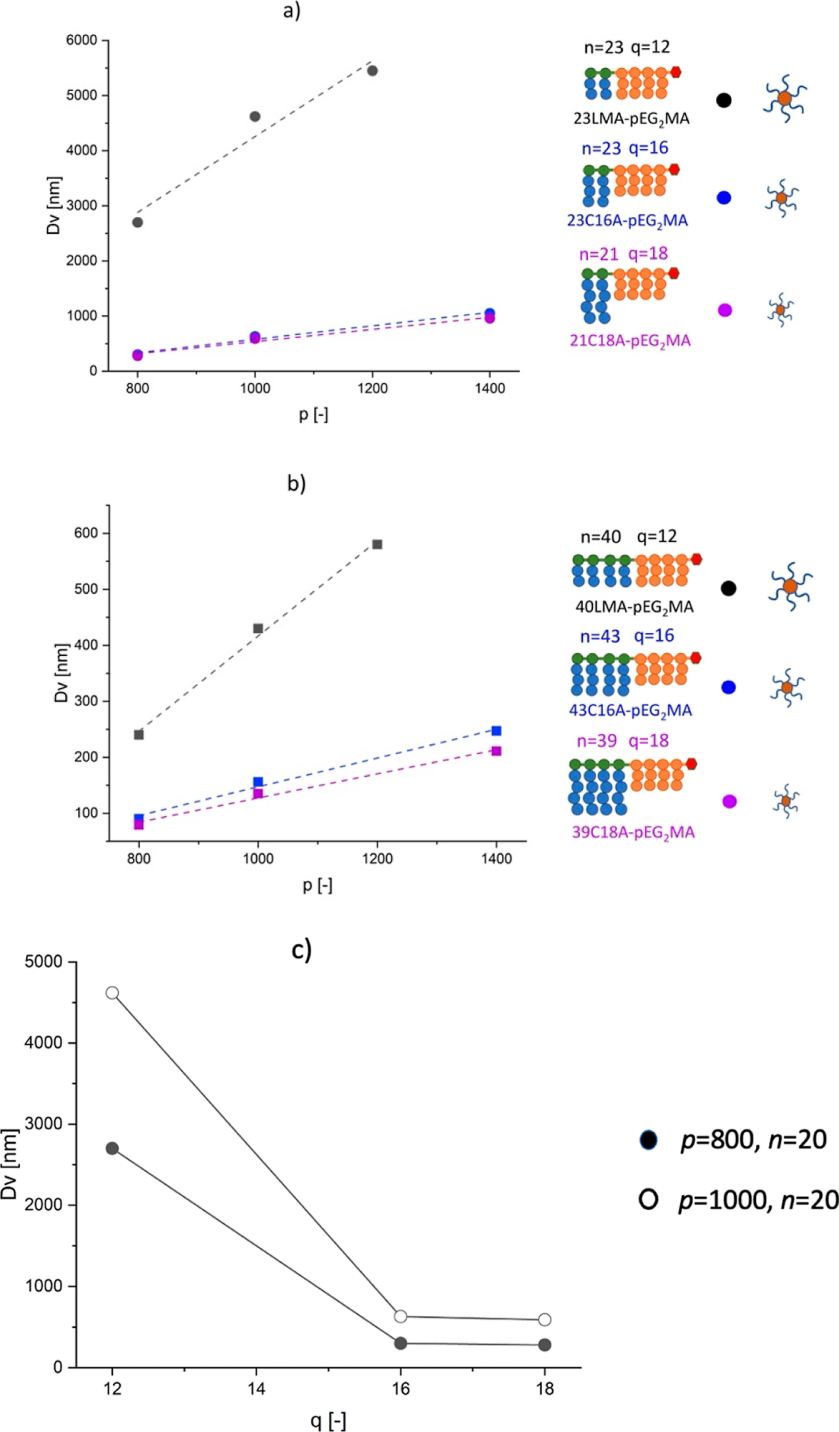
Volume-average size of the NPs formed by the block copolymers in dectol at a fixed temperature of 25 °C, below the *T*_cp_, as a function of three parameters: *n*, *p*, and *q*. (a) Comparison between copolymers with the same *n* (*n* = 20, circles) at different *p* (shown on the *x* axis) and *q* (*q* = 12 in black, *q* = 16 in blue, and *q* = 18 in purple). The dashed lines represent the linear fits of the experimental data, with *R*^2^ = 0.950 (*q* = 12), *R*^2^ = 0.985 (*q* = 16), and *R*^2^ = 0.981 (*q* = 18); (b) comparison between copolymers with the same *n* (*n* = 40, squares) at different *p* (shown on the *x* axis) and *q* (*q* = 12 in black, *q* = 16 in blue, and *q* = 18 in purple). The dashed lines represent the linear fits of the experimental data, with *R*^2^ = 0.995 (*q* = 12), *R*^2^ = 0.990 (*q* = 16), and *R*^2^ = 0.990 (*q* = 18); (c) comparison between copolymers with equal *n* (*n* = 20, circles) and different *p* (*p* = 800, full symbol and *p* = 1000, empty symbol) at different lengths *q* of the solvophilic monomer (shown on the *x* axis). The data for the cases with *q* = 12 are reproduced from ref (42). Copyright 2023 American Chemical Society.

From this equation, the nanoparticle diameter, with a fixed stabilizing agent, grows linearly with *p*, a behavior that was verified experimentally as shown in Figure 6a,b independently from *n* and *q* .

When the value of *n* is fixed, an increase in the number of units of the thermo-responsive block leads to the formation of bigger nanoparticles, whose diameter increases linearly with *p* in all the cases considered. This behavior supports the geometrical model suggested in eq 4. On the other hand, the slope of these linear trends differs in the various cases. This implies that *A*_cov_ is actually a function of the solvophilic block microstructure. In particular, the slope of *D*_v_ vs *p* is steeper for short solvophilic blocks, leading us to conclude that *A*_cov_ is directly proportional to *n*. As a matter of fact, at fixed values of *p*, the size of the nanoparticles has an inverse dependence on *n*, as confirmed by comparing *D*_v_ in Figure 6a,b.

In addition to the role played by *n*, it is also possible to disclose the dependence of the nanoparticle size on *q*, which represents the length of the brushes of the stabilizing block. As shown in Figure 6c, when all the other parameters are fixed, an increase in *q* leads to a decrease in the nanoparticle average diameter, suggesting better stabilizing properties of the solvophilic block when *q* is increased. Interestingly, we noticed that the influence of *q* on *D*_v_ is particularly evident when moving from 12 to 16. On the other hand, it reaches a plateau for *q* equal to 16 and 18, in perfect agreement with the observation already made for *T*_cp_.

**Figure 7:**
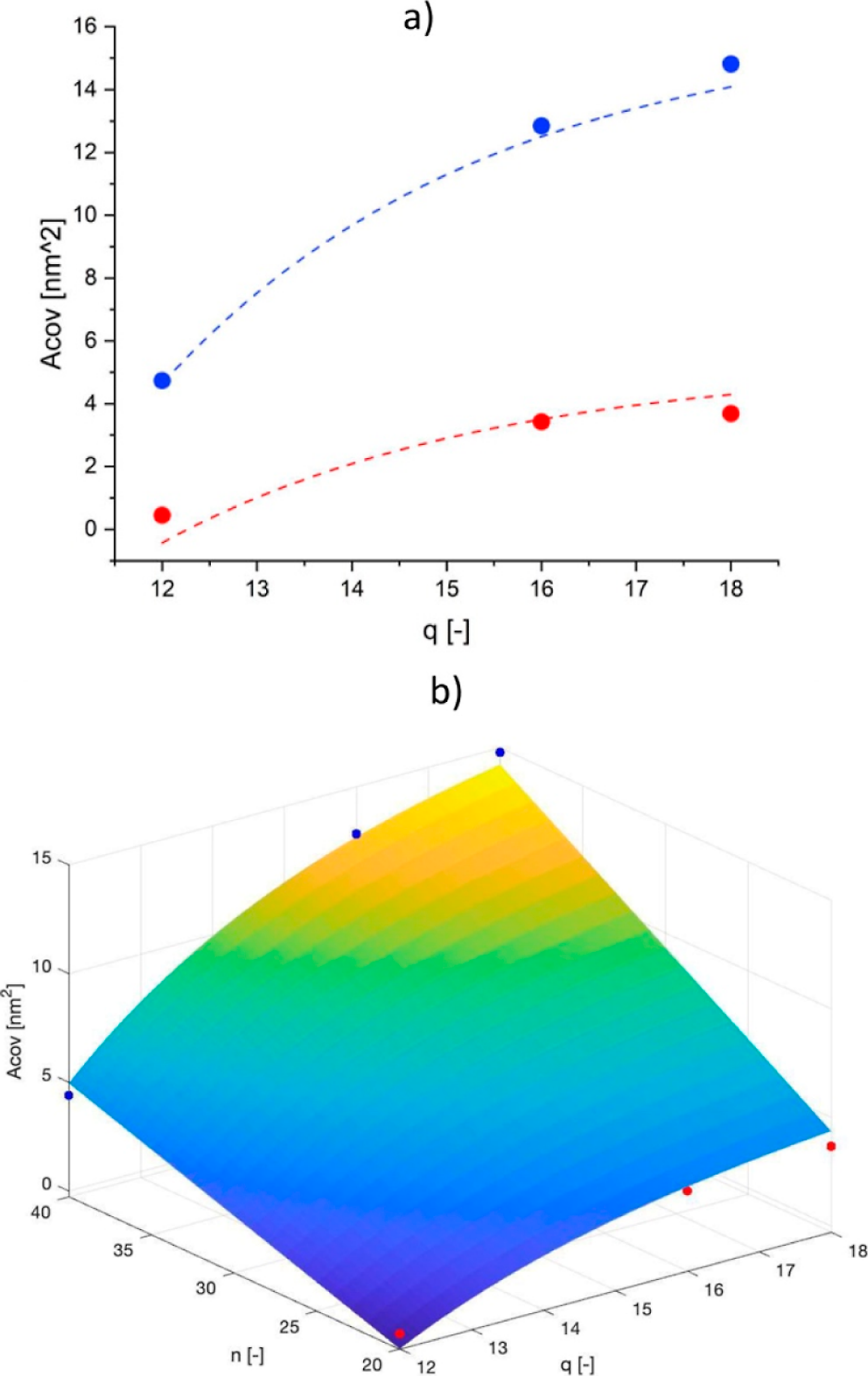
(a) *A*_cov_ as a function of the structural parameters of the solvophilic block, namely, *n* (*n* = 20, blue dots and *n* = 40, red dots) and *q* (shown on the *x* axis). The dashed lines represent the fitting given by eq 6; (b) 3D graph obtained through MATLAB relating *A*_cov_ to n and p based on the best fitting function (eq 6) and comparison with the experimental data. The data for the cases with *q* = 12 are reproduced from ref (42). Copyright 2023 American Chemical Society.

To shed light on the dependence of *A*_cov_ on both *n* and *q*, this parameter was computed for all the polymer nanoparticles through eq 5 and shown as a function of *n* and *q* in Figure 7a. Indeed, *A*_cov_ is not significantly affected by the number of thermo-responsive units *p* (at fixed *n* and *q*), whose effect is only a change in *D*_v_, which compensates the role of *p* in eq 5. Therefore, a mean value of *A*_cov_ for samples with fixed *n* and *q* and different *p* can be considered.
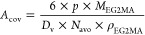
5

From the figure, it is possible to observe that an increase in *n* increases significantly the area covered by each single solvophilic block on the nanoparticle surface. This evidence may indicate that the solvophilic block is not extended in the continuous phase, as it is typically imagined, but rather disposed tangentially to the surface, so that its extension defines also the area occupied on the nanoparticle surface itself. The same reasoning applies to *q*, whose increment at fixed *n* leads to a wider surface area covered by the stabilizing block. The impact of this parameter on the surface area is more accentuated at larger *n*. In fact, while at *n* = 20 *A*_cov_ reaches a plateau for *q* > 16, at *n* = 40 *A*_cov_ still increases moving from *q* = 16 to *q* = 18.

This functional relationship highlighted for *A*_cov_ with *n* and *q* has been expressed through eq 6, which was used to fit the experimental data through a genetic algorithm aimed at minimizing the RSS (eq 7). In particular, we hypothesized an exponential dependence on *q* to justify the asymptotic behavior of *A*_cov_ with respect to this parameter, and a linear dependence on *n* to account for the increase in the surface area covered by copolymers with longer stabilizing blocks.
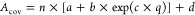
6
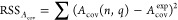
7

The best set of parameters found was [0.54 −9.30 −0.29 −5.50], leading to a , representative of a good agreement with the experimental data in the range of parameters examined in this study, as shown by the dashed lines in Figure 7a.

The design space for *A*_cov_ as a function of *n* and *q* is shown in Figure 7b, where the predictions of the models have been compared to the actual data, showing a good agreement.

This confirms that it is possible to predict the *A*_cov_ and therefore the stability of the block copolymer simply by changing the structural parameters of the solvophilic block, namely, *n* and *q*. This information, coupled with the length of the solvophobic block *p*, further allows us to determine a priori the size of the final nano-object through eq 4, giving an important tool to obtain well-defined nano-objects according to easily modifiable parameters. In addition, the possibility of controlling *A*_cov_ by acting only on *n* and *q* allows us to leave *p* for the fine tuning of *T*_cp_, thus providing a way for decoupling these two important properties for a thermo-responsive formulation.

Overall, we demonstrated that it is possible to synthesize copolymers with well-defined physico-chemical properties and thermo-responsive behavior by exploiting the living nature of RAFT polymerization to control a wide range of microstructural parameters such as length of the solvophilic and thermo-responsive block, length of the brushes of the macroCTA, and solid content. This paves the way to the development of novel and highly controlled thermo-responsive systems in non-polar solvents.

## 4 Conclusions

In this work, RAFT polymerization was exploited to produce a library of modular diblock copolymers with a thermo-responsive behavior in a non-polar medium, dectol. The living nature of this controlled radical polymerization allowed synthesizing copolymers with a well-defined structure by simultaneously controlling four parameters, namely, the length of the solvophilic and thermo-responsive blocks (*n* and *p*), the length of the brushes (*q*) of the solvophilic block, and the solid content.

The tuning of these parameters is a valuable strategy to control key features of the nano-objects formed in dectol. In particular, it was found that there is a linear correlation between the degree of polymerization of the thermo-responsive block and the cloud point of the copolymers. In addition, although typically disregarded, we demonstrated that the microstructure of the solvophilic block also affects the *T*_cp_, with a linear and an exponential dependence on *n* and *q*, respectively. Therefore, the role played by the stabilizer in dictating the thermal response of the copolymer cannot be neglected during its design. This is even more important considering that *n* and *q* strongly influence the morphology of the nano-objects formed in dectol and the area covered by each single chain on the nanoparticle surface.

Indeed, *A*_cov_ grows linearly with *n* and, to a certain extent, with *q*. This may indicate that the stabilizer is disposed tangentially with respect to the surface, covering it with the alkyl side chains.

Although the effects of *p* have been extensively characterized for different core-forming blocks and solvents, the role played by *n* and *q* in affecting the morphology and size of polymer nanoparticles is often neglected. However, the results reported in this work show that they are equally important in dictating the behavior of the final block copolymers. This also provides a way for decoupling the main properties of the formulation, such as *T*_cp_ and nanoparticle size. A rational design of the copolymer microstructure, involving parameters specific for both the solvophilic and solvophobic portions, is therefore crucial for developing materials with very specific properties and suitable for advanced applications. Indeed, polymeric nano-objects in non-polar media have already been successfully applied as oil emulsifiers,^6^ water shut-off systems,^52^ and lubricant additives.^23^ The addition of the thermo-responsive behavior to these traditional materials would further expand their scope, allowing these fields to advance toward previously unimaginable horizons, as it already happened in the biomedical field, where thermo-responsive polymers are acting as the main drivers for innovation in tissue engineering and drug delivery.

## Supporting Information Available

GPC and NMR characterizations of the macroCTAs and of the block copolymers, summary of the properties of the different copolymers synthesized at different solid contents (including *T*_cp_), HPLC analysis of commercially available EG_2_MA, average size, polydispersity, and *A*_cov_ of the spherical NPs synthesized, and visualization of the 4 behaviors used to generate the phase diagrams (PDF)
