# Supplementary material for: Role of the Polymer Microstructure in Controlling Colloidal and Thermo-Responsive Properties of Nano-Objects Prepared Via RAFT Polymerization in a Non-polar Medium
Source: Langmuir. 2023 Jul 12;39(29):10133–44. doi: 10.1021/acs.langmuir.3c01065 (PMC10373479; doi:10.1021/acs.langmuir.3c01065)
Supplement: Supplementary file 1 — la3c01065_si_001.pdf [file la3c01065_si_001.pdf]

Supporting information for

Role of the Polymer Microstructure in Controlling Colloidal and Thermo-Responsive Properties of Nano-Objects Prepared via RAFT Polymerization in a Non-Polar Medium

Gianmaria Gardoni, Nicolò Manfredini, Giulia Bagnato, Mattia Sponchioni*, Davide Moscatelli

Department of Chemistry, Materials and Chemical Engineering "Giulio Natta", Politecnico di Milano, via Mancinelli 7, 20131 Milano, Italy

Correspondence to: mattia.sponchioni@polimi.it

The two lipophilic monomers produced through acylation of alkyl alcohols were analyzed via NMR after the purification process explained in Section 2. As it can be seen from **Figure S1** and **Figure S2**, the excess of methacryloyl chloride has been removed through the work-up, leaving only the product and the unconverted alcohol. In particular, it was possible to calculate the alcohol conversion in each case by using **Equation S1**.

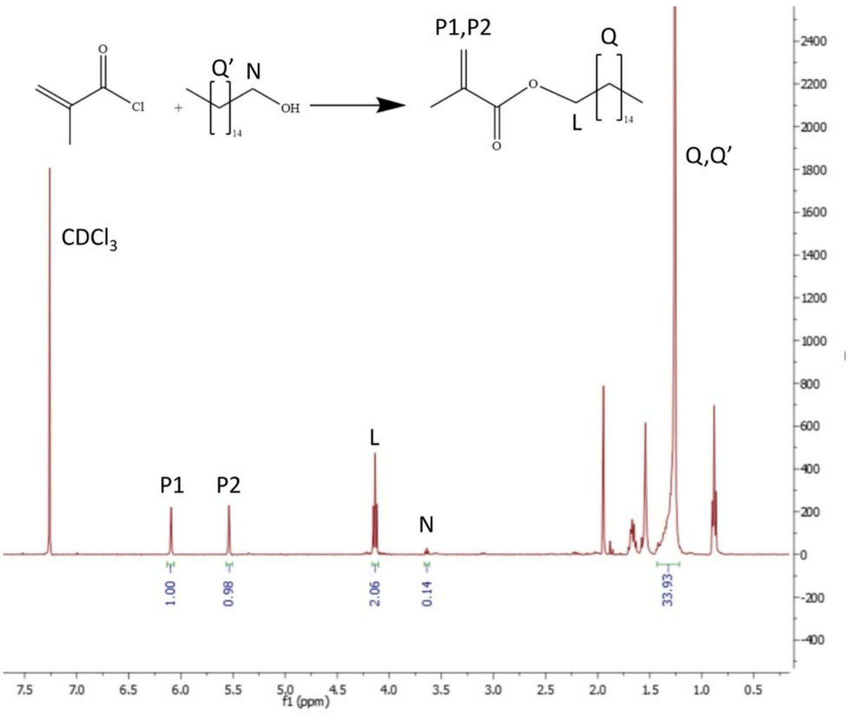

Figure S1 - ^{1}H NMR spectrum of C16A after purification.

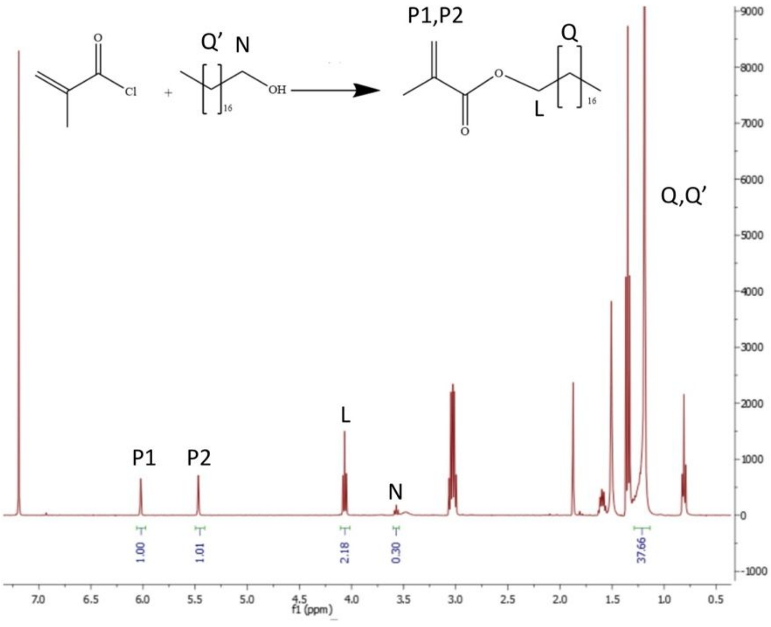

Figure S2 - ^{1}H NMR spectrum of C18A after purification.

$$\chi_{alcohol} = 1 - \frac{N}{N+L} \tag{S1}$$

The synthesized monomers were employed for the synthesis of four different macroCTAs, which differ for the length of the brushes (C16A or C18A) and the number of repeating units (n=20 or 40). The four macroCTAs were analyzed via NMR to determine the monomer conversion and the actual degree of polymerization n through **Equation S2** and **Equation S3**.

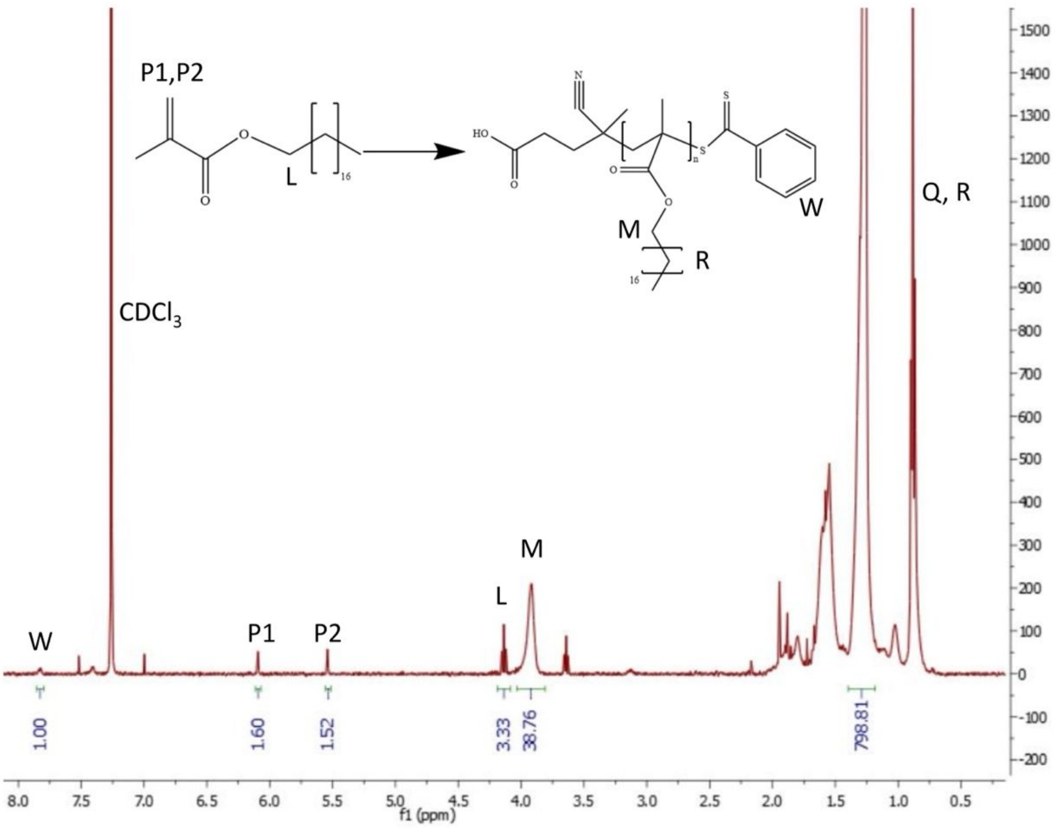

Figure S3 – ^{1}H NMR spectrum of nC18A with n=40 before the purification step. The sample was dissolved in deuterated chloroform ($CDCl_3$) and analyzed on a Bruker 400 MHz spectrometer.

$$\chi_{monomer} = 1 - \frac{L}{(L+M)} \tag{S2}$$

$$n = \frac{M}{W} \tag{S3}$$

Where L is associated to the area of the protons close to the methacrylate group in the unconverted monomer, M to the area of the protons of the repeating unit incorporated in the polymer and W refers to the area of the signal of two hydrogens in the aromatic ring of the RAFT agent, respectively.

The macroCTAs were further analyzed via GPC to determine the molecular weight distribution. From **Figure S4**, it can be noticed that all of the polymers have a narrow distribution and an increasing molecular weight according to the degree of polymerization *n* targeted, easily modifiable by acting on the ratio between the monomer and the RAFT agent concentrations.

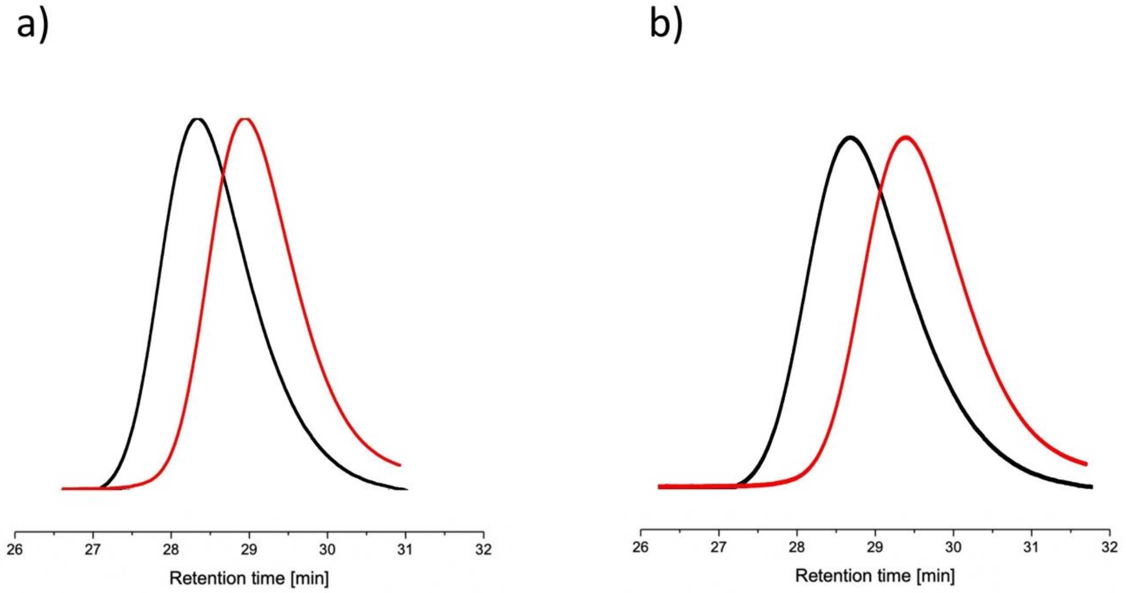

Figure S4 - GPC chromatogram of the a) nC16A and b) nC18A macroCTAs synthesized with n=20 (red curve) and n=40 (black curve). The peaks shift to lower retentions times with increasing n, as expected.

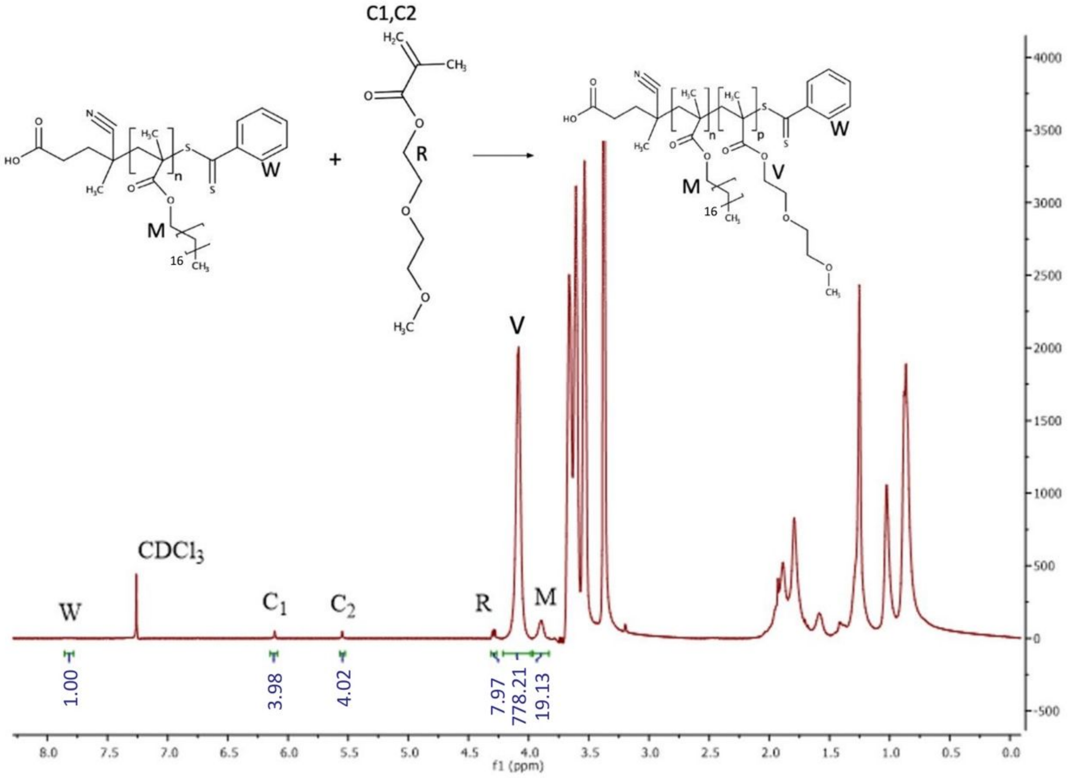

Figure S5 – ^{1}H NMR spectrum of 21C18A-800EG$_2$MA.

The EG_2MA conversion and the degree of polymerization p of the thermo-responsive block were calculated via ^{1}H-NMR through **Equation S4** and **Equation S5**:

$$\chi_{EG2MA} = 1 - \frac{R}{(R+V)} \tag{S4}$$

$$p = \frac{V}{W} \tag{S5}$$

Where V represents the peak area associated to the two hydrogens close to the ester bond in the EG_2MA incorporated in the polymer, W represents the peak area associated to the two hydrogens of the aromatic ring of the RAFT agent and R refers to the area associated to the two hydrogens close to the ester bond in the unconverted EG_2MA.

The CTA efficiency was calculated according to **Equation S6**.

$$\text{Eff}_{CTA} = \frac{DP_{target}}{p} * \chi_{EG2MA} \tag{S6}$$

Table S1 - Monomer conversion (χ) and p obtained via ^{1}H NMR and Mn, Mw and Đ measured via GPC for the nC16A-pEG_2MA diblock copolymers synthesized at 20% w/w.

20% w/w Syntheses	**χ_{EG2MA} [%]**	***p* [-]**	**Eff_{CTA} [%]**	**Mn [Da]**	**Mw [Da]**	***Đ* [-]**
23C16A-800EG_2MA	99	825	97	161660	216620	1.34
23C16A-1000EG_2MA	99	1064	93	210660	290710	1.38
23C16A-1400EG_2MA	96	1501	90	278510	401050	1.44
43C16A-800EG_2MA	96	855	90	177740	232840	1.31
43C16A-1000EG_2MA	94	1194	80	251170	344100	1.37
43C16A-1400EG_2MA	91	1378	93	319130	424440	1.33

Table S2 - Monomer conversion (χ) and p obtained via 1H NMR and Mn, Mw and Đ measured via GPC for the nC16A-pEG2MA diblock copolymers synthesized at 30% w/w.

30% w/w Syntheses	**χ_{EG2MA} [%]**	***p* [-]**	**Eff_{CTA} [%]**	**Mn [Da]**	**Mw [Da]**	***Đ* [-]**
23C16A-800EG$_2$MA	99	741	107	151840	206500	1.36
23C16A-1000EG$_2$MA	99	982	100	191190	271490	1.42
23C16A-1400EG$_2$MA	98	1463	94	243110	364670	1.5
43C16A-800EG$_2$MA	99	849	93	165800	235440	1.42
43C16A-1000EG$_2$MA	95	1095	87	228930	277000	1.21
43C16A-1400EG$_2$MA	92	1358	95	299200	421870	1.41

Table S3 - Monomer conversion (χ) and p obtained via 1H NMR and Mn, Mw and Đ measured via GPC for the nC16A-pEG2MA diblock copolymers synthesized at 40% w/w.

40% w/w Syntheses	**χ_{EG2MA} [%]**	***p* [-]**	**Eff_{CTA} [%]**	**Mn [Da]**	**Mw [Da]**	***Đ* [-]**
23C16A-800EG$_2$MA	99	778	102	149140	213260	1.43
23C16A-1000EG$_2$MA	98	1089	90	205673	306450	1.49
23C16A-1400EG$_2$MA	98	1395	98	276170	461200	1.67
43C16A-800EG$_2$MA	97	832	93	183620	244210	1.33
43C16A-1000EG$_2$MA	97	1024	95	241580	299560	1.24
43C16A-1400EG$_2$MA	96	1412	95	303670	403880	1.33

Table S4 - Monomer conversion (χ) and p obtained via 1H NMR and Mn, Mw and Đ measured via GPC for the nC18A-pEG2MA diblock copolymers synthesized at 20% w/w.

20% w/w Syntheses	**χ_{EG2MA} [%]**	***p* [-]**	**Eff_{CTA} [%]**	**Mn [Da]**	**Mw [Da]**	***Đ* [-]**
21C18A-800EG$_2$MA	99	786	102	159570	215410	1.35
21C18A-1000EG$_2$MA	99	1122	89	218100	307510	1.41
21C18A-1400EG$_2$MA	99	1507	92	275680	396990	1.44
39C18A-800EG$_2$MA	99	753	106	184530	252800	1.37
39C18A-1000EG$_2$MA	99	1100	91	261660	371550	1.42
39C18A-1400EG$_2$MA	99	1428	97	356600	570560	1.6

Table S5 - Monomer conversion (χ) and p obtained via 1H NMR and Mn, Mw and Đ measured via GPC for the nC18A-pEG2MA diblock copolymers synthesized at 30% w/w.

30% w/w Syntheses	**χ_{EG2MA} [%]**	***p* [-]**	**Eff_{CTA} [%]**	**Mn [Da]**	**Mw [Da]**	***Đ* [-]**
21C18A-800EG$_2$MA	95	897	85	170387	231726	1.36
21C18A-1000EG$_2$MA	95	1128	84	204263	302309	1.48
21C18A -1400EG$_2$MA	98	1369	100	285754	380053	1.33
39C18A-800EG$_2$MA	98	783	100	187490	283110	1.51
39C18A-1000EG$_2$MA	99	1121	89	282890	364930	1.29
39C18A-1400EG$_2$MA	94	1437	91	375130	521430	1.39

Table S6 - Monomer conversion (χ) and p obtained via 1H NMR and Mn, Mw and Đ measured via GPC for the nC18A-pEG2MA diblock copolymers synthesized at 40% w/w.

40% w/w Syntheses	χ_{EG2MA} [%]	*p* [-]	Eff_{CTA} [%]	Mn [Da]	Mw [Da]	*Đ* [-]
21C18A -800EG$_2$MA	96	871	88	175280	243639	1.39
21C18A-1000EG$_2$MA	95	1051	90	218754	308443	1.41
21C18A-1400EG$_2$MA	93	1484	87	264111	409372	1.55
39C18A-800EG$_2$MA	97	760	102	191640	241470	1.26
39C18A-1000EG$_2$MA	95	956	99	286940	375890	1.31
39C18A-1400EG$_2$MA	96	1394	97	371990	543110	1.46

The presence of small early-eluting shoulders in the GPC traces of the block copolymers was attributed to the presence of some ethylene glycol dimethacrylate (EGDMA) impurities in the commercial EG$_2$MA available. To verify this, a HPLC analysis has been performed to compare the elution times of both commercial EG$_2$MA and EGDMA, used as reference, as shown in **Figure S6**. The separation was performed on a Restek C18 resin (250*4.6 mm, with 5 µm particles). The mobile phase consists of a mixture of 70% v/v deionized water and 30% v/v acetonitrile, kept constant for 5 min. The acetonitrile content was then increased to 100% v/v from 5 to 20 minutes. The flow rate was kept constant at 1 mL/min, the detector lamp was set to 219 nm and the oven temperature to 35°C.

The presence of a second peak in the commercial EG$_2$MA, with a retention time equal to the one of EGDMA confirms the initial assumption of dimethacrylate impurities.

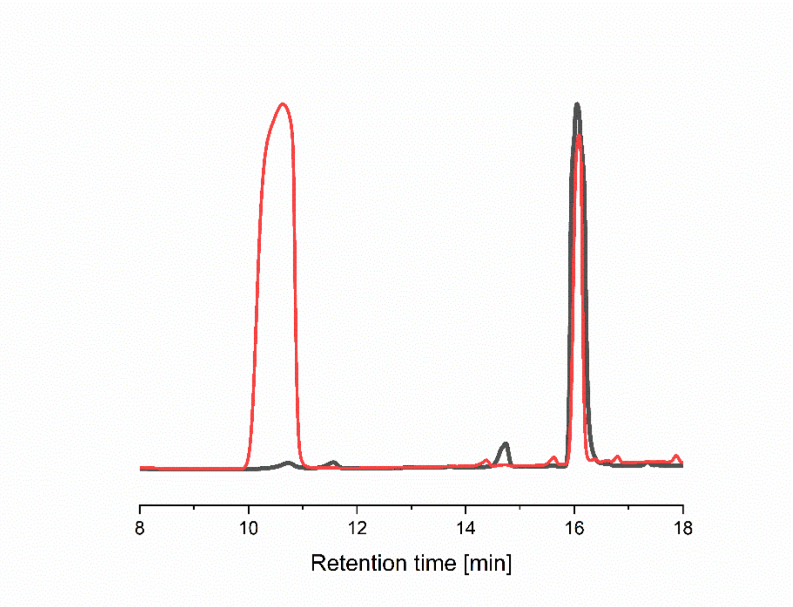

Figure S6 – HPLC chromatogram of commercially available EG_2MA (red curve) and EGDMA (black curve).

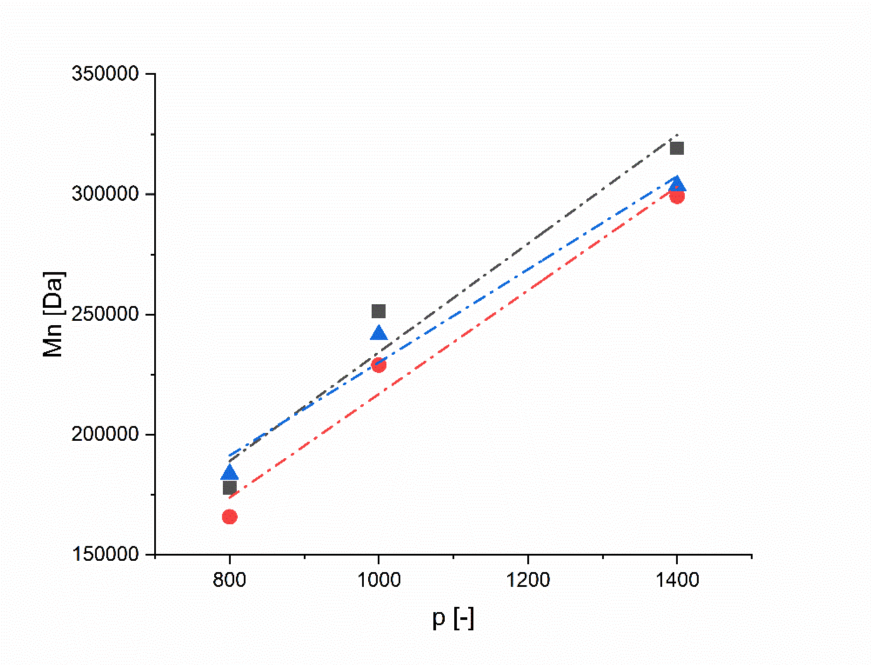

Figure S7 - Mn vs p for the 43C16A-pEG2MA 20% (black squares), 30% (red circles) and 40% (blue triangles) copolymers. The dashed lines show the linear fittings of the experimental data, with R^2=0.955 (20% syntheses), R^2=0.975 (30% syntheses) and R^2=0.971 (40% syntheses)

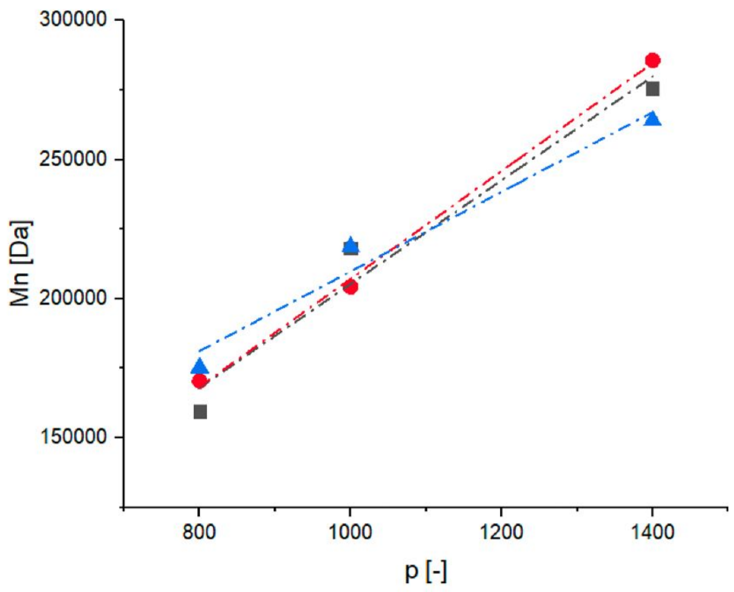

Figure S8 - Mn vs p for the 21C18A-pEG2MA 20% (black squares), 30% (red circles) and 40% (blue triangles) copolymers. The dashed lines show the linear fittings of the experimental data, with R^2=0.963 (20% syntheses), R^2=0.998 (30% syntheses) and R^2=0.969 (40% syntheses)

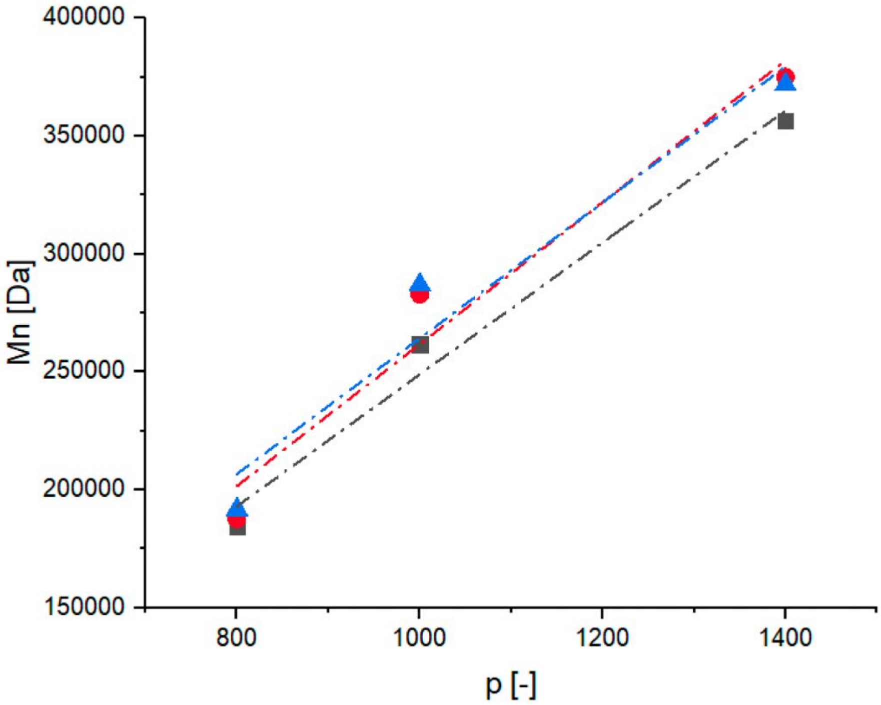

Figure S9 - Mn vs p for the 39C18A-pEG2MA 20% (black squares), 30% (red circles) and 40% (blue triangles) copolymers. The dashed lines show the linear fittings of the experimental data, with R^2=0.983(20% syntheses), R^2=0.961 (30% syntheses) and R^2=0.951 (40% syntheses)

Table S7 - Tcp values for the copolymers synthesized. The cloud points for the 40LMA-pEG$_2$MA samples are taken from [1] .

Sample	**T_{cp} [˚C]**
23LMA-400EG$_2$MA	59
23LMA-600EG$_2$MA	62
23LMA-800EG$_2$MA	71
23LMA-1200EG$_2$MA	81
40LMA-400EG$_2$MA	55
40LMA-600EG$_2$MA	57
40LMA8000EG$_2$MA	62
40LMA-1200EG$_2$MA	72
23C16A-800EG$_2$MA	67
23C16A-1000EG$_2$MA	71
23C16A-1400EG$_2$MA	82
43C16A-800EG$_2$MA	53
43C16A-1000EG$_2$MA	59
43C16A-1400EG$_2$MA	69
21C18A-800EG$_2$MA	65
21C18A-1000EG$_2$MA	72
21C18A-1400EG$_2$MA	83
39C18A-800EG$_2$MA	52
39C18A-1000EG$_2$MA	60
39C18A-1400EG$_2$MA	72

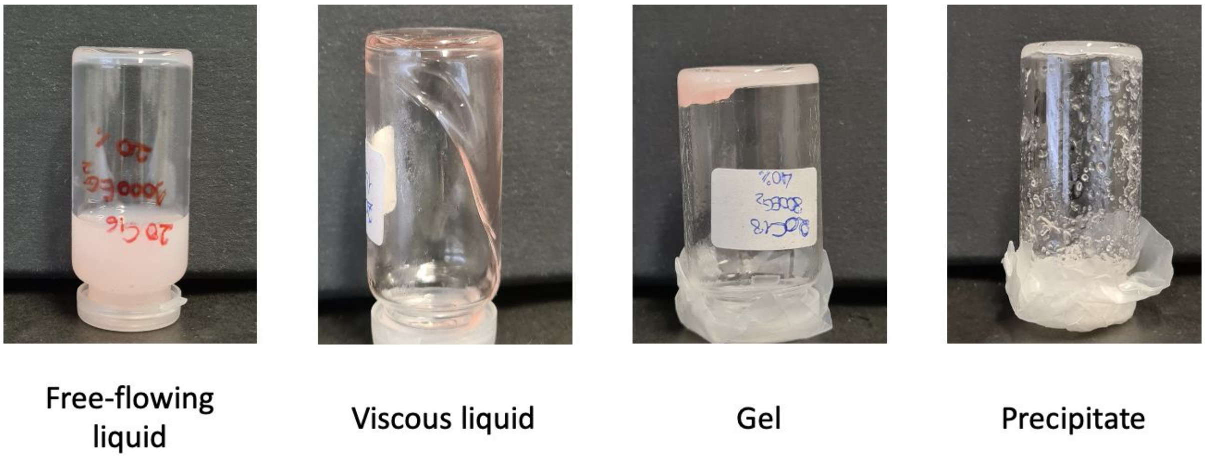

Figure S10 - Visual appearance of samples in the different regions of the phase diagram at a fixed temperature (25°C). From left to right: 23C16A-1000EG2MA at 20% w/w; 23C16A-800EG2MA at 40% w/w; 23C18A-800EG2MA at 40% w/w and 23C18A-1400EG2MA at 40% w/w.

Table S8 - Volume-average diameter, PDI and A_{cov} for the copolymers able to form spherical nanoparticles at temperatures below cloud point (i.e. 25 °C). The data for the 40LMA-pEG_2MA samples based on LMA are taken from [1] .

20% w/w Syntheses	D_v [nm]	PDI [-]	A_{cov} [nm^2]
23LMA-800EG_2MA	2700	0.22	0.54
23LMA-1000EG_2MA	4620	0.21	0.40
23LMA-1200EG_2MA	5450	0.17	0.40
40LMA-800EG_2MA	240	0.19	6.12
40LMA-1000EG_2MA	430	0.10	4.28
40LMA-1200EG_2MA	580	0.21	3.80
23C16A-800EG_2MA	300	0.22	4.90
23C16A-1000EG_2MA	630	0.16	2.92
23C16A-1400EG_2MA	1050	0.19	2.45
43C16A-800EG_2MA	90	0.17	16.34
43C16A-1000EG_2MA	160	0.18	11.78
43C16A-1400EG_2MA	250	0.12	10.42
21C18A-800EG_2MA	280	0.19	5.25
21C18A-1000EG_2MA	590	0.17	3.11
21C18A-1400EG_2MA	960	0.23	2.68

39C18A-800EG$_2$MA	80	0.11	18.62
39C18A-1000EG$_2$MA	140	0.19	13.62
39C18A-1400EG$_2$MA	210	0.13	12.20

References

1. Gardoni, G., Manfredini, N., Monzani, M., Sponchioni, M. & Moscatelli, D. Thermoresponsive Modular Nano-Objects Via RAFT Dispersion Polymerization in a Non-Polar Solvent. (2022) doi:10.1021/acsapm.2c01598.
